# STUDENTS HELPING OLLI BUILD A MORE DIVERSE MEMBERSHIP WITH AN ASSESSMENT OF AVAILABLE TRANSPORTATION OPTIONS

**DOI:** 10.1093/geroni/igae098.0184

**Published:** 2024-12-31

**Authors:** Laura Donorfio

**Affiliations:** University of Connecticut, Waterbury, Connecticut, United States

## Abstract

An undergraduate adulthood and aging class partnered with Osher Lifelong Learning Institute’s (OLLI) Diversity Committee to identify transportation resources available for seniors in their community to potentially increase the diversity of the OLLI membership. A needs assessment research framework was used to put a semester long, four-phase research project into place, each with specific goals and milestones. General milestones included researching available transportation resources and organizations for seniors; documenting target audiences and associated difficulties using said resources; exploring potential solutions and providing recommendations for future pilot projects. The Director of the OLLI program attended monthly report outs to assist in the creation of a doable pilot project, helping identify key constraints and logistics. The semester ended with a formal research report out, executive summary, and the university career counselor attending to help students brainstorm and create solid resume entries. The presentation will finish by describing what was learned via best practices and practices to avoid, particularly intergenerational “gaps” that surfaced on how to collect research, the types of research, and the breadth and depth of the research. Lastly lessons learned as a teacher on how to be in the “middle” of both generations and the OLLI director to help make the project a success and how to get student “buy in” by continually reviewing the importance of participating in a service-learning class project for future career aspirations.

